# Dual immobilization of magnetite nanoparticles and biosilica within alginate matrix for the adsorption of Cd(II) from aquatic phase

**DOI:** 10.1038/s41598-022-15844-w

**Published:** 2022-07-06

**Authors:** Mahdi Safari, Reza Rezaee, Reza Darvishi Cheshmeh Soltani, Esrafil Asgari

**Affiliations:** 1 Environmental Health Research Center, Research Institute for Health Development, Kurdistan University of Medical Sciences, Sanandaj, Iran; 2 Department of Environmental Health Engineering, Faculty of Health, Kurdistan University of Medical Sciences, Sanandaj, Iran; 3 Department of Environmental Health Engineering, School of Health, Arak University of Medical Sciences, Arak, Iran; 4 Department of Environmental Health Engineering, School of Public Health, Khoy University of Medical Sciences, Khoy, Iran

**Keywords:** Environmental chemistry, Biotechnology, Environmental sciences

## Abstract

The adsorption of cadmium ions by magnetite (Fe_3_O_4_)@biosilica/alginate (MBA nano-hybrid) was the main aim of the present investigation. Herein, MBA nano-hybrid was synthesized via chemical precipitation technique. As-synthesized MBA nano-hybrid was characterized using FT-IR, FESEM and XRD analyzes. Based on the results, the maximum adsorption capacity of the adsorbent for the removal of Cd(II) was obtained at the initial pH of 7.0. At the initial Cd(II) concentration of 40 mg/L, increasing the reaction time to 180 min led to the Cd adsorption of 35.36 mg/g. Since the removal of Cd(II) after the reaction time of 60 min was insignificant, the reaction time of 60 min was considered as optimum reaction time for performing the experimental runs. According to the results, Langmuir isotherm and pseudo-second order kinetic models were the best fitted models with high correlation coefficients (R^2^ > 0.99). The results of thermodynamic study indicated exothermic (positive ΔH°) and spontaneous nature (negative ΔG°) of the adsorption of Cd(II) on the surface of MBA nano-hybrid. Negligible reduction in the adsorption capacity of the nano-hybrid was observed (16.57%) after fifth experimental runs, indicating high reusability potential of the as-synthesized nano-hybrid adsorbent.

## Introduction

Pollution of water resources due to the uncontrolled discharge of industrial wastewaters has become a global health and environmental concern^1^. Heavy metals are among the most important pollutants in industrial effluents due to their characteristics such as non-degradability, durability, aggregation, and toxicity^2–4^. Cadmium, chromium, copper, zinc, and nickel are among the most common heavy metals available in high concentrations in industrial wastewaters^5,6^.

Cadmium can enter aqueous environment from various sources including non-ferrous metals production, electroplating of metals, battery manufacturing, paint industry, photography, and lead and zinc mines. In addition, volcanic eruptions and forest fires are among the most important natural sources of cadmium entry into the environment^1,7,8^. Among the heavy metals, much attention is paid to cadmium, due to high toxicity and being categorized as Group B1 (probable human carcinogen compound) by the U.S. Environmental Protection Agency (U.S. EPA)^9,10^. Cadmium can be bio accumulated like other heavy metals causing cancer even at lower concentrations^7^. Studies showed that exposure to various concentrations of cadmium can lead to acute and chronic complications in the kidney, liver, nervous, and cardiovascular systems^1^. The World Health Organization (WHO) has established a maximum concentration level (MCL) of 0.003 mg/L for cadmium in potable water^11^.

Due to the health problems associated with the presence of cadmium in water, development of effective methods for the removal of cadmium is crucial. Various treatment methods have been utilized to eliminate cadmium ions from water including chemical precipitation, membrane technologies, biological processes, electrochemical techniques, flotation, ion exchange, and adsorption process^11^. Among the aforementioned methods, the absorption process is considered among researchers as an economic and appropriate process for the elimination of heavy metal ions from liquid phase due to its simplicity, reliability, and safety^2,11,12^. Various adsorbents such as activated carbon, clay, natural zeolite, furnace ash, bentonite, polymers, resin, biological absorbents, and hazelnut shells have been used to remove heavy metals from aqueous environments^13,14^. Silica materials are considered as desirable adsorbents because of the ability of their surface functional groups in the adsorption of target pollutants^15,16^. Diatomaceous earth, also called biological silica (biosilica), is a siliceous sedimentary rock containing more than 90% silicon dioxide. Because of the presence of active surface groups on high surface area, biological silica is considered as a suitable adsorbent for the adsorption of various organic and inorganic pollutants from wastewater^17,18^. Among different synthetic adsorbents, nano-sized adsorbents have gained much attention due to their fine size and consequently large surface areas for the efficient adsorption of target pollutants^19,20^. Magnetite nanoparticles are proposed as common adsorbent owing to their large specific surface area and ease of synthesis to be used as individual adsorbent or in combination with other adsorbents^1,7,14^. In addition, easy separation of magnetite nanoparticles by an external magnetic field is the main advantage of this adsorbent. However, the adsorption capacity of nanoparticles and their selectivity are not satisfactory as well as their stability in acidic conditions^21,22^.

The separation and recycling of the adsorbent are of important problems during the implementation of nanstructured absorbents. Thus, the immobilization of nanoparticles within a suitable matrix is proposed as an effective approach to enhance reusability potential of the adsorbent. Polymeric substances such as alginate and chitosan are the most extensively used compounds to immobilize nanostructured adsorbents. Among polymeric substances, alginate is attracted more attentions among researchers because of its high reactivity, low cost and high adsorption capacity^23^. Environmentally, alginate is nontoxic with high biodegradability^24^. Therefore, it can be widely used as a gel matrix, membrane material, and water-blocking agent. Due to its high quantities of carbonyl groups and oxygen atoms, alginate is an efficient adsorbent for metal ions^24^. Therefore, the current study focused on the preparation of magnetite (Fe_3_O_4_)@biosilica/alginate (MBA) nano-hybrid and its application for the adsorption of cadmium ions from aqueous aqueous environments. Dual immobilization of magnetite nanoparticles and biosilica was considered to avoid the washout of fine particles from the liquid phase. This approach was utilized to not only effectively immobilize nanostructured adsorbents but also improve the adsorption capacity of the immobilized components. In addition to high adsorption capacity, the aforementioned nanostructured adsorbents can be removed from the solution and being recycled by an external magnetic field.

## Materials and experimental procedure

### Materials

Diatomaceous earth (~ 97.5% SiO_2_ basis) and sodium alginate (C_6_H_9_NaO_7_) were purchased from Sigma Aldrich Company. Cadmium nitrate (Cd (NO_3_)_2_) and other chemicals were provided by Merck Co. (Germany). The chemicals used in the current study were reagent and laboratory grade and utilized without any changes.

### Preparation of nano-adsorbents

#### Preparation of magnetic biosilica

In the current study, the common chemical precipitation method by FeCl_2_.4H_2_O, FeCL_3_.6H_2_O, and biosilica were added to ammonia solution under nitrogen gas flow to prepare magnetic biosilica via a simple chemical precipitation method. Firstly, 2.0 g of biosilica was added to the distilled water (100 mL) and placed in an ultrasonic bath for 12 h to attain a homogeneous mixture with weigh percent of 2%. Also, 0.8457 g of iron chloride (II) and 2.2992 g of iron chloride (III) were dissolved in distilled water (50 mL). In the following, 50 mL of the biosilica mixture was added to 50 mL of iron chloride solutions and stirred for 30 min under nitrogen gas flow of 80 °C. Then, 25 mL of 25% ammonia (w/w) was injected into the stirring mixture under nitrogen gas flow for a further 1.0 h. In the next step, the magnetic biosilica was collected using a Block Neodymium Magnet and washed with ethanol and distilled water repeatedly. Finally, as-synthesized magnetic biosilica was dried in an oven at 70 °C^25^.

#### Preparation of MBA nano-hybrid

To prepare the MBA nano-hybrid, 1.0 g of sodium alginate (C_6_H_9_NaO_7_) was added to 100 mL of acetic acid 1.0 M and mixed. Then, 1.0 g of magnetic biosilica was also added to acetic acid and sodium alginate solution, and then mixed until obtaining a homogenous viscous solution. The mixture was subsequently provided in a final volume of 100 mL (4.0:1.0 ratio for 15% NaOH to 95% ethanol). The viscous mixture of sodium alginate, alginate/magnetic biosilica was dropped into NaOH/ethanol using a syringe. The bullet-shaped compounds formed in this stage were set aside for 24 h. The beads obtained from nano-hybrid were washed with the pure water until reaching neutral pH then dried at room temperature. Finally, the dried beads were crushed in mortar and graded using a standard sieve^26^.

#### Experimental procedure

In the current study, all the experiments were done in batch flow mode reactors using 100-mL glass Erlenmeyers. Also, the effect of the main parameters of solution pH (2.0–9.0), contact time (10–180 min), MBA nano-hybrid dosage (0.25–4.0 g/L), cadmium concentration (10–320 mg/L), and temperature (25–55 °C) on the adsorption process of Cd(II) ions were assessed. For preparation of stock solution (1000 mg/L of Cd), double distilled water was used. The solution pH was adjusted using either 0.1 M NaOH or 0.1 M H_2_SO_4_. The certain concentrations of cadmium (50.00 mL) were prepared and the desired changes were applied to each Erlenmeyer. The batch reactors were then placed on a shaker (150 rpm) to mix and carry out the adsorption process. After the completion of the process, Cd-contained samples were taken from the reactor at regular time intervals and analyzed. The kinetics studies, isotherm models, thermodynamics, recovery of nano-hybrid and the effect of other cations (Na^+^, K^+^, Mg^2+^ and Ca^2+^) on the adsorption of Cd(II) were also studied. The experiments were performed thrice and the mean values were recorded. The adoptions efficiency of Cd(II) ions was calculated according to Eq. (1), in which C_0_ and C_e_ are the initial concentration and equilibrium concentrations of Cd(II) ion (mg/L), respectively:

1 $$\% Removal = \frac{{C_{0} - C_{e} }}{{C_{e} }} \times 100$$

The quantity of Cd(II) ions adsorbed onto the nano-hybrid in equilibrium at time t (min) was determined Eq. (2)^27^:

2 $$q_{t} = \frac{{(C_{0} - C_{t} ) V }}{W}.$$

#### Analyses

The Fourier transform infrared spectroscopy (FT-IR), scanning electron microscopy (SEM), and X-ray diffraction (XRD) techniques were used to identify and characterized the nano-hybrid. The morphology of the nano-hybrid was investigated with SEM (Model: MIRA III, TESCAN, Czech Republic). The crystalline structure of nano-hybrid was examined using a XRD apparatus (Model: PW-1730, Philips, the Netherland). Additionally, the surface functional groups of the nano-hybrid were determined using the FT-IR spectroscopy (Model: AVATAR, Thermo, USA). For this analysis, the KBr pellet was prepared using specific amount of the powder sample. Then, as-prepared pellet was used in the instrument to obtain the spectrum in the wavenumber range of 500–4000 cm^-1^.

The cadmium amount was measured in the samples before and after the adsorption process using atomic adsorption spectrometry (AAS, Model: AA-7000, Shimadzu, Japan)^2^.

### Consent for publication

All the authors agree to publish this article.

## Results and discussion

### Nano-hybrid characterization

**Figure 1:**
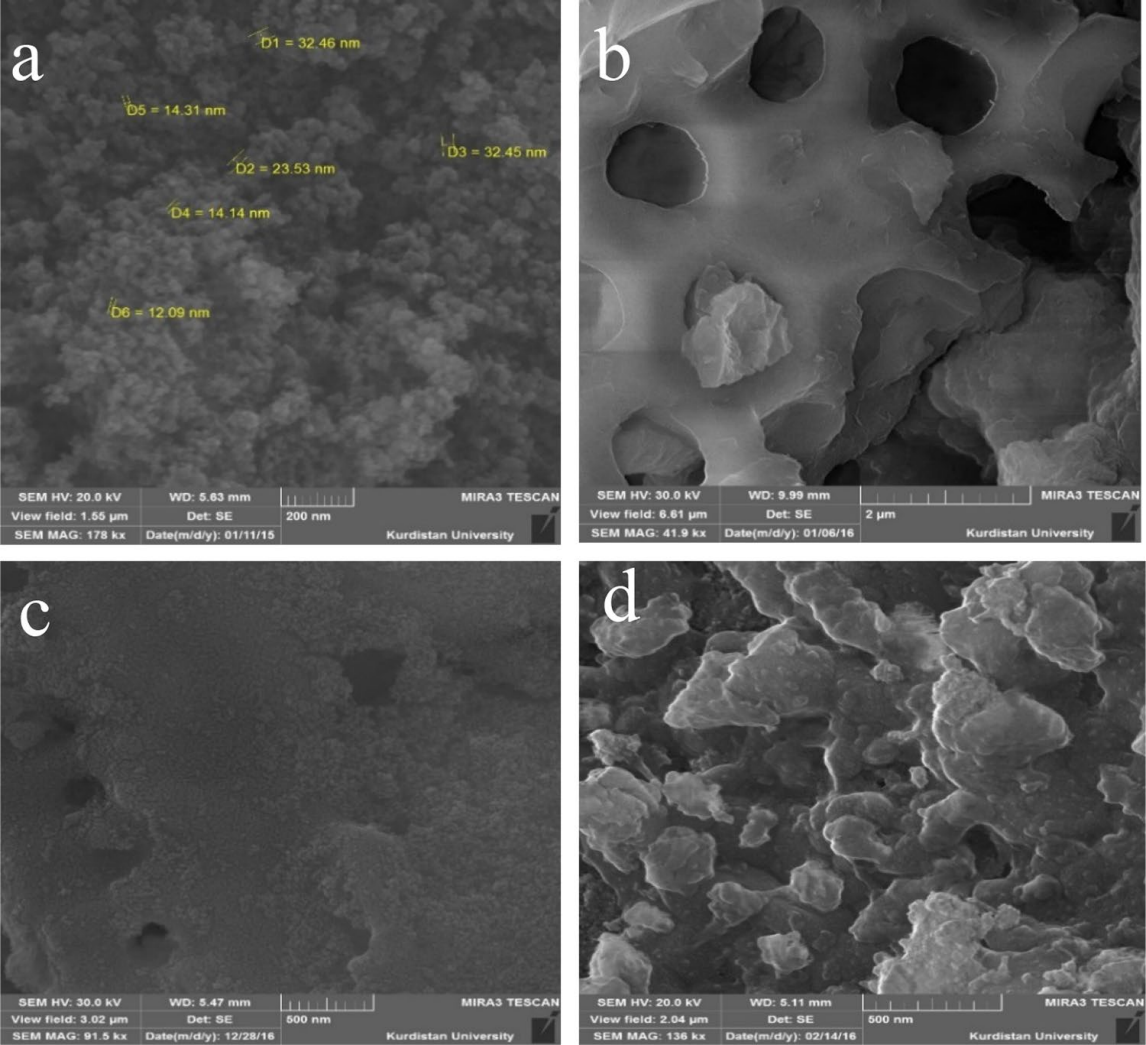
SEM images of nano-sized Fe_3_O_4_ (**a**), biosilica (**b**), biosilica/Fe_3_O_4_ (**c**) and MBA nano-hybrid (**d**).

Figure 1 shows SEM images of the synthesized nano-hybrid. Comparing the SEM images shows significant change in the morphology of the nano-hybrid after the immobilization.

Figure 1a shows surface morphology of the magnetite particles. As shown, magnetite particles are formed in nano-size during the synthesis procedure. Porous structure of biosilica can be observed in Fig. 1b, indicating its suitability to be used as support for the magnetite nanoparticles. Figure 1c shows that magnetite nanoparticles are distributed on the biosilica surface. Biosilica surface is covered by ultrafine particles of magnetite. Figure 1d displays biosilica/magnetite immobilized into alginate matrix. The appropriate entrapment of biosilica/magnetite in the alginate matrix is obvious. However, the nano-hybrid has suitable porous structure containing biosilica and especially magnetite nanoparticles exposed to the target pollutant ions for the efficient adsorption.

Although the size of the nano-hybrid increased in comparison with the composing nanostructured components but such combination, as explained in the adsorption experiments section, resulted in the enhanced adsorption of Cd ions. On the other hand, the nature of the MBA nano- hybrid makes it a superior adsorbent for the adsorption of heavy metal ions such as Cd.

**Figure 2:**
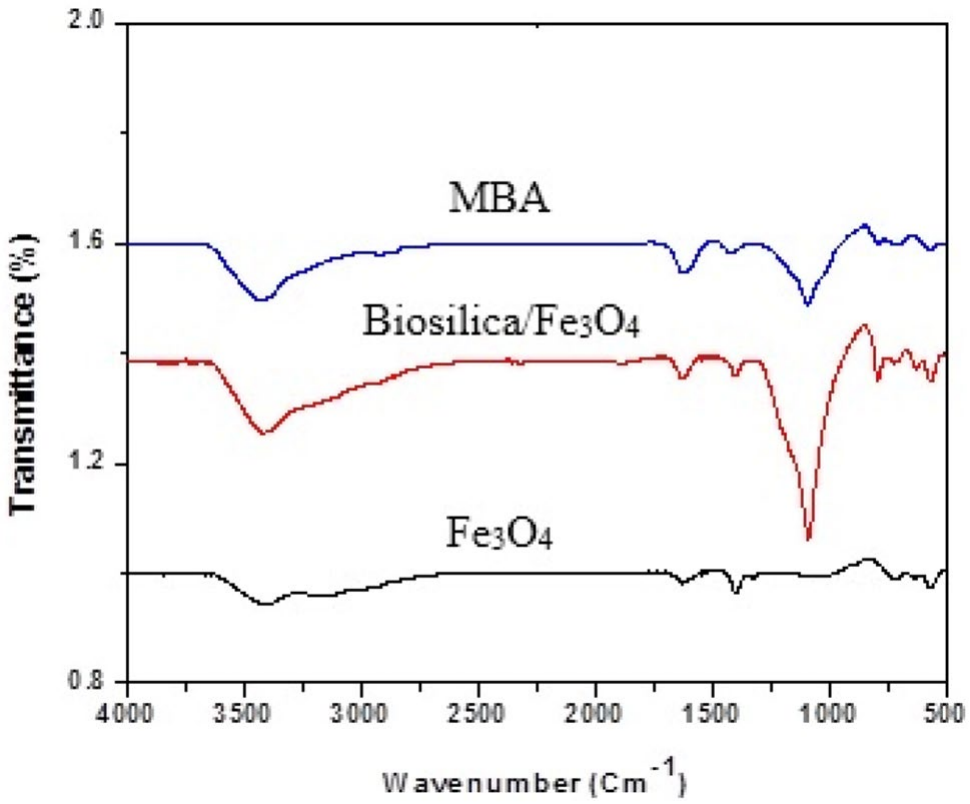
FT-IR spectra of Fe_3_O_4_, biosilica/Fe_3_O_4_ and MBA nano-hybrid.

The effect of adding biosilica and alginate on the functional groups of Fe_3_O_4_ was studied using the FT-IR analysis. Figure 2 presents the spectra of Fe_3_O_4_, Biosilica/Fe_3_O_4_ and MBA nano-hybrid. A new peak placed at 1200 cm^-1^ (symmetric stretching mode of Si–O-Si bond) reveals the presence of biosilica in the structure of biosilica/Fe_3_O_4_; however, the intensity of biosilica characteristic peak was reduced due to the presence of alginate in the nano-hybrid. Moreover. the peak located at wavenumber of 800 cm^-1^ represents stretching Si–O group of the biosilica structure^16^. The weak peak placed around 570 cm^-1^ indicates the presence of Fe_3_O_4_.

The peak around 3400 cm^-1^ may be associated with the stretching vibration of OH group, which intensified after the immobilization of Fe_3_O_4_ nanoparticles, indicating the involvement of hydroxyl group in the immobilization of Fe_3_O_4_ nanoparticles^26^.

**Figure 3:**
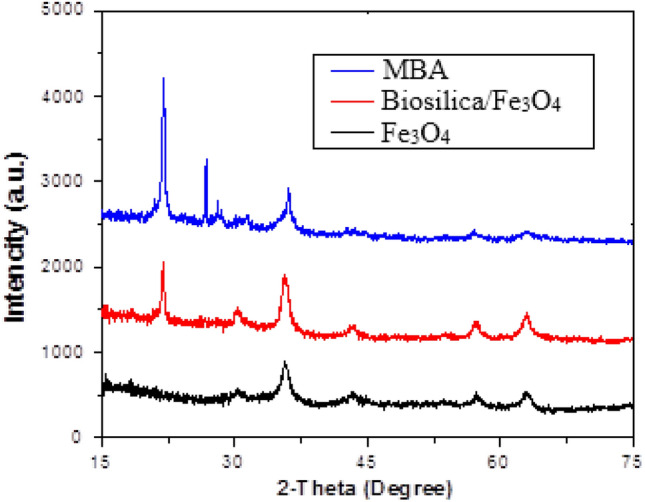
The XRD pattern of Fe_3_O_4_, biosilica/Fe_3_O_4_ and MBA nano-hybrid.

The crystalline structure of the nano-hybrid was analyzed using XRD analysis. The patterns are provided in Fig. 3. It is obvious that the combination of biosilica with Fe_3_O_4_ resulted in appearing new peak at 21.9°. It is in accordance with the JCPDS Card number of 00–001-0647 which is associated with the pure silica structure^28^. The third pattern (MBA) confirmed crystalline nature of the nano-hybrid even after the immobilization via amorphous alginate polymeric matrix^29^.

Moreover, intensity of the peak located at 30° increased, which demonstrated the appropriate incorporation of Fe_3_O_4_ into the biosilica structure. On the other hand, alginate biopolymer not only resulted in appearing a new peak at 25° but also slightly displaced the biosilica peak. It indicated the enlargement of the basal spacing of the biosilica due to the interactions between biosilica and alginate^29^. The XRD patterns confirmed the presence of alginate, biosilica and Fe_3_O_4_ in an integrated lattice.

### Effect of experimental parameters

#### Effect of solution pH

**Figure 4:**
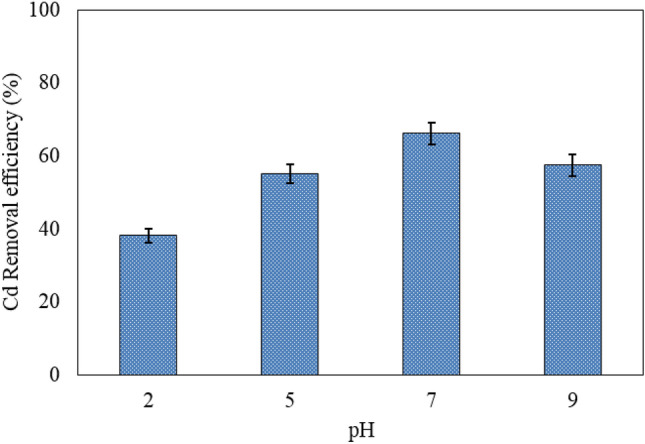
Effect of pH on the adsorption of Cd on MBA nano-hybrid. ([Cd]_0_ = 40 mg/L, [MBA]_0_ = 1.0 g/L, temperature = 25 °C, contact time = 60 min).

The adsorption of target pollutants especially heavy metal ions on a hybrid adsorbent such as MBA nano-hybrid usually depends on the initial pH of the solution^30^. The hydrolysis and deposition properties of metal ion species are influenced by the solution pH^31^. In this study, the effect of initial pH on the adsorption of Cd ions was investigated at the initial Cd concentration of 40 mg/L, nano-hybrid dosage of 1.0 g/L, temperature of 25 °C, stirring rate of 150 rpm and contact time of 60 min (Fig. 4).

The MBA nano-hybrid showed less affinity to Cd ions at strong acidic conditions. The alginate in the structure of MBA nano-hybrid contains large amounts of polysaccharides, some of which are linked to proteins and other components. These macromolecules on the surface of the alginate have several surface functional groups such as carboxyl, thiol, amine, and phosphate^31^.

**Figure 5:**
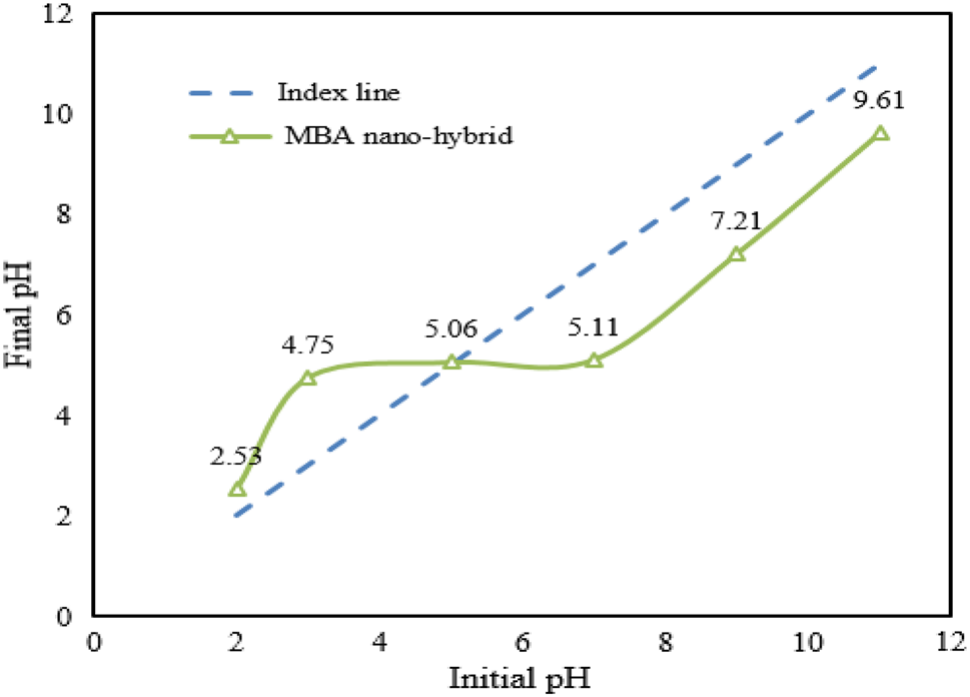
pH_zpc_ of MBA nano-hybrid.

The efficiency of Cd adsorption depends on the protonation and deprotonation of the surface unctional groups of the nano-hybrid surface. The nano-hybrid electric charge and ionic forms of Cd ions depend on solution pH. To understand the type and amount of the surface charge of the studied MBA nano-hybrid at different pHs, pH_zpc_ value of the nano-hybrid was measured. Based on the result, pH_zpc_ value of about 5.06 was obtained. Therefore, the surface charge of the nano-hybrid was negative at pH values above 5.06. At pH < pH_zpc_, the surface charge was positive (Fig. 5).

The highest adsorption efficiency of Cd ions was obtained at the initial pH of 7.0. Increasing the initial pH from 7.0 to 9.0 led to a 9.0% reduction in the removal efficiency of Cd ions. In fact, the gravity force between the positive Cd ions and the MBA nano- hybrid reached the highest at pH values above 5.0. The lowest Cd removal value was observed at pH 2.0, which is due to the competition between Cd ions and H^+^ ions for occupying adsorptive sites of the nano-hybrid.

In addition, the results of other studies show that in most cases, maximum Cd removal occurred in the pH range of 4.0–7.0 (weak acidic and neutral), which is in accordance with the results of the present study^32^. Maximum Cd removal occurred in the studies of Bayramoglu et al. in pH 6.0^31^, Alakhras in pH 5.0^33^, Dolgormaa et al. in pH 5.0^34^ and Shen et al. in pH 6.0^32^.

#### Effect of contact time

**Figure 6:**
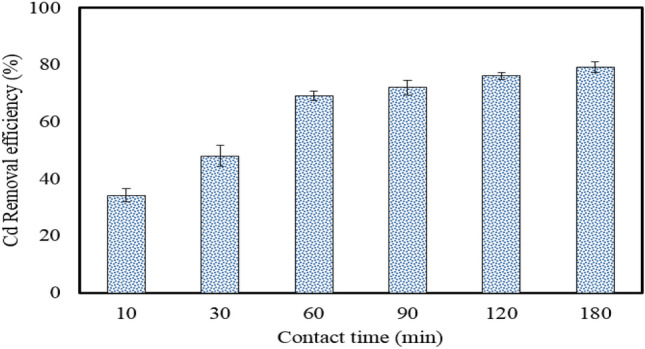
The effect of adsorption time on the removal of Cd by the MBA nano-hybrid. ([Cd]_0_ = 40 mg/L, pH = 7.0, adsorbent = 1.0 g/L, temperature = 25 °C).

After determining the optimum pH, the effect of contact time on the removal of Cd by the MBA nano-hybrid was investigated at the initial Cd concentration of 40 mg/L, adsorbent dose of 1.0 g/L and temperature of 25 °C within the contact time of 180 min. As shown in Fig. 6, the increase in contact time promoted Cd removal efficiency. The removal efficiency of Cd increased from 34.20 to 79.23% by increasing the contact time from 10 to 180 min, respectively.

At the beginning of the adsorption process, the removal efficiency was fast, reaching 69.17% and then almost reached a plateau at later times. The major reason for this behavior is the saturation of the active sites in the nano-hybrid, which do not allow for further adsorption. This can be explained by the fact that initially the number of sites on the surface of the nano-hybrid is very high, which makes adsorption very fast, but over time, the active sites become saturated, hence reducing adsorption rate^35^.

#### Effect of initial Cd concentration

**Figure 7:**
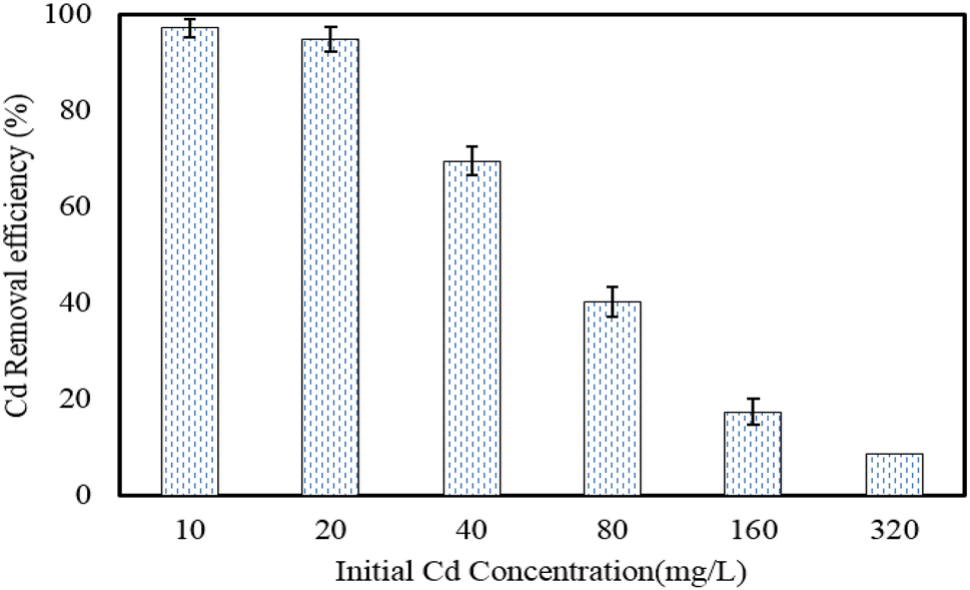
The effect of initial concentration of Cd on its removal by the MBA nano-hybrid (pH = 7.0, adsorbent = 1.0 g/L, temperature = 25 °C, time = 60 min).

As displayed in Fig. 7, it is observed that increasing the initial Cd concentration results in the decreased removal efficiency of Cd by the nano-hybrid. As can be seen in Fig. 7, the removal efficiency decreased from 97.3 to 8.57% with increasing the concentration of Cd from 10 to 320 mg/L, respectively. At the initial Cd concentration of 40 mg/L, the removal efficiency was 69.57%.

As the adsorption sites are constant for a certain amount of nano-hybrid, the removal efficiency of Cd(II) decreases with increasing the Cd(II) concentration. The decrease in the ratio of Cd(II) residue to initial concentration of Cd(II) by increasing the initial concentration of Cd(II) can also be ascribed to the enhancement in the driving force due to an increase in the initial Cd(II) concentration, which is caused by the repulsive forces between Cd(II) and prevention of adsorption of Cd(II) on the nano-hybrid.

A study by Benhima et al.^36^ on the removal of both Cd(II) and Pb ions from aqueous solution via the adsorption on microparticles of dry plants and another study by Wang et al.^37^ on the removal of Cd (II) by crude clay (raw attapulgite clay) from aqueous solution have shown the decreased removal efficiency by increasing the initial concentration of the target pollutants. This finding could be due to the number of fixed sites on a given amount of adsorbent.

#### Effect of adsorbent dosage

**Figure 8:**
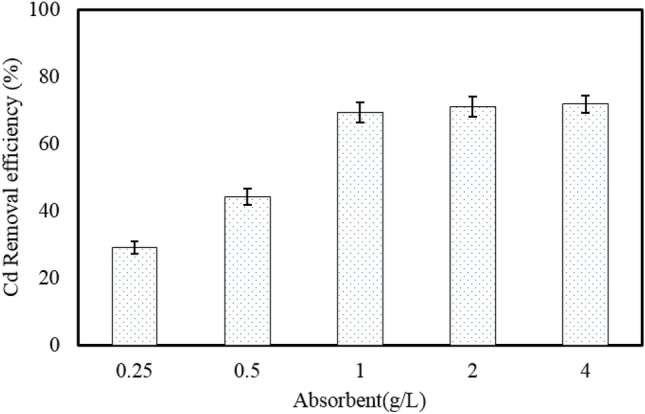
The effect of dosage of adsorbent on the removal efficiency of Cd(II) by MBA nano-hybrid (C_0_ = 40 mg/L, pH = 7.0, temperature = 25 °C, time = 60 min).

As can be noted in Fig. 8, Cd removal efficiency increases with increasing dosage of nano-hybrid. Increasing the adsorbent dosage from 0.25 to 1.0 g/L improved the adsorption efficiency of Cd(II) ions from 29.1 to 69.3%, respectively. Also, increasing the nano-hybrid dosage from 2.0 to 4.0 g/L had no significant effect on Cd(II) ion removal efficiency and resulted in a very slight increase in the efficiency from 71.1 to 71.8%, respectively. It is clear that as the amount of adsorbent increases, the number of available adsorption sites increases, so the adsorption efficiency improves, but the adsorption rate which is the amount of pollutant adsorbed per mass unit of the adsorbent (mg/g), diminished. Therefore, the adsorbent dosage of 1.0 g/L was selected as the optimal dosage for performing the rest of experiments^35,37^.

#### Kinetic study

The prediction of the adsorption process is one of the major factors during designing an adsorption system^38^. Four different kinetic models including pseudo-first-order, pseudo-second order, intra-particle diffusion and Elovich models (Eqs. (3) to (6)) were used to investigate the kinetic of Cd adsorption on the MBA nano-hybrid at an initial concentration of 40 mg/L.

The pseudo-first and pseudo-second order kinetic models are expressed in the Eqs. (3) to (4)^30,39^:

3 $$\ln \left( {q_{e} - q_{t} } \right) = Lnq_{e} - k_{1} t$$

4 $${\raise0.7ex\hbox{$t$} \!\mathord{\left/ {\vphantom {t {q_{t} }}}\right.\kern-\nulldelimiterspace} \!\lower0.7ex\hbox{${q_{t} }$}} = {\raise0.7ex\hbox{$1$} \!\mathord{\left/ {\vphantom {1 {k_{2} q_{e}^{2} }}}\right.\kern-\nulldelimiterspace} \!\lower0.7ex\hbox{${k_{2} q_{e}^{2} }$}} + {\raise0.7ex\hbox{$t$} \!\mathord{\left/ {\vphantom {t {q_{e} }}}\right.\kern-\nulldelimiterspace} \!\lower0.7ex\hbox{${q_{e} }$}}$$

where *q*_*t*_ and *q*_*e*_ (*mg/g*) are the values of absorbed cadmium at *t* and equilibrium time, respectively. *K*_*1*_* (1/min)* and *K*_*2*_* (g/(mg.min))* are the rate constants of the first- and second-order models, respectively.

The intra-particle diffusion model (Eq. (5)) was also used to characterize the multi-step and competitive adsorptions in the adsorption process, and the Elovich model (Eq. (6)), based on the adsorption capacity, was employed to describe the kinetics of chemical adsorption^40^.

5 $${\text{q}}_{{\text{t}}} = {\text{k}}_{int} {\text{t}}^{1/2} + {\text{ C}}$$

where *K*_*int*_ is the intra-particle infiltration rate constant in mg.min^1^^/^^2^/g, *C* is the intra-particle infiltration constant in mg/g and *q*_*t*_ is as previously defined.

6 $$q_{t} = {\raise0.7ex\hbox{$1$} \!\mathord{\left/ {\vphantom {1 \beta }}\right.\kern-\nulldelimiterspace} \!\lower0.7ex\hbox{$\beta $}}{\text{Ln}}\left( {\alpha \beta } \right) + {\raise0.7ex\hbox{$1$} \!\mathord{\left/ {\vphantom {1 \beta }}\right.\kern-\nulldelimiterspace} \!\lower0.7ex\hbox{$\beta $}}{\text{Ln}}t$$

where *α* is the initial absorption rate as mg/g.min, and *β* is the surface coverage (desorption) as g/mg. In the Elovich equation, the values of α and β parameters was obtained by plotting *q*_*t*_ against *Lnt*.

**Figure 9:**
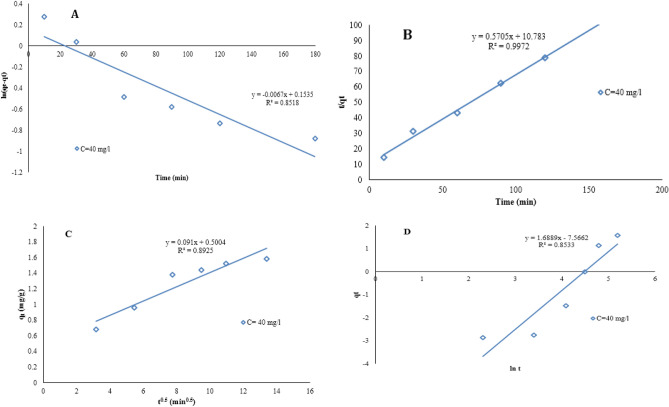
The kinetic models for the removal of Cd by MBA nano-hybrid: (**a**) Pseudo-first order, (**b**) Pseudo-second order, (**c**) Intra-particle (**d**) and Elovich models (pH = 7.0, adsorbent = 1.0 g/L, temperature = 25 °C, optimum time = 60 min).

The results of kinetic study are summarized in Table 1 and Fig. 9. Table 1 shows that the pseudo-second-order kinetic model with the correlation coefficient of 0.99 is the most suitable kinetic model to describe the adsorption process and the calculated adsorption capacity (q_cal_) is completely consistent with the experimental data (q_exp_).

**Table 1:** Kinetic parameters for the removal of Cd by the MBA nano-hybrid. (pH = 7.0, [MBA]_0_ = 1.0 g/L, temperature = 25 °C, contact time = 60 min).

Kinetic models	Kinetic parameters	Values at C_0_ = 40 mg/L
Pseudo-first order	q_exp_ (mg/g)	35.38
	q_e_ (mg/g)	22.16
	K_1_(1/min)	0.0067
	r^2^	0.8518
Pseudo-second order	q_e_ (mg/g)	35.44
	K_2_(g/(mg.min))	0.12
	r^2^	0.9972
Intra-particle	C (mg/g)	0.5
	K_int_ (mg/g.min)	0.091
	r^2^	0.89
Elovich	α (mg/g.min)	27.5
	β (g/mg)	0.59
	r^2^	0.85

Given the low regression coefficients of the pseudo-first-order kinetic models, intra-particle diffusion and Elovich models with the regression coefficients of 0.85, 0.89 and 0.85, respectively, these models cannot describe the Cd adsorption process on the MBA nano-hybrid well and the mentioned adsorption system does not follow these models. Therefore, the pseudo-second-order kinetic model is more suitable for the Cd adsorption process on the MBA nano-hybrid. Badruddoza et al. in their study of heavy metal removal with Fe_3_O_4_/cyclodextrin polymeric nanocomposite showed the highest agreement with the pseudo-second-order model and the regression coefficient was above 99%^41^. The results of the present study are also in agreement with those of Yi et al.^42,43^.

### Isotherms study

Adsorption isotherm shows how the heavy metal ions are distributed between the liquid phase and the solid phaseat the equilibrium state^42^. To further elucidate the mechanism of Cd(II) adsorption, Langmuir, Freundlich, and Temkin isotherm models were used [Eqs. (7) to (9)]^27,44,45^.

7 $$q_{e} = \frac{{q_{m} K_{L} c_{e} }}{{1 + K_{L} c_{e} }}$$

8 $$q_{e} = K_{F} c_{e}^{\frac{1}{n}}$$

9 $$q_{e} = {\text{B Ln }}K_{t} + B Ln c_{e}$$

In these equations, *C*_*e*_ is the equilibrium concentration of the adsorbed material (mg/L), *q*_*e*_ is the adsorption capacity at equilibrium (mg/g), *q*_*m*_ is the maximum adsorption capacity (mg/g), *K*_*L*_ is the Langmuir equilibrium constant (L/mg). In the Freundlich isotherm model, *K*_*F*_ is the Freundlich constant (mg/g) and 1/n is the absorption intensity. In the Temkin isotherm model, B is the isotherm constant (J/mol) and A is the binding constant of Temkin (L/g)^44^.

**Figure 10:**
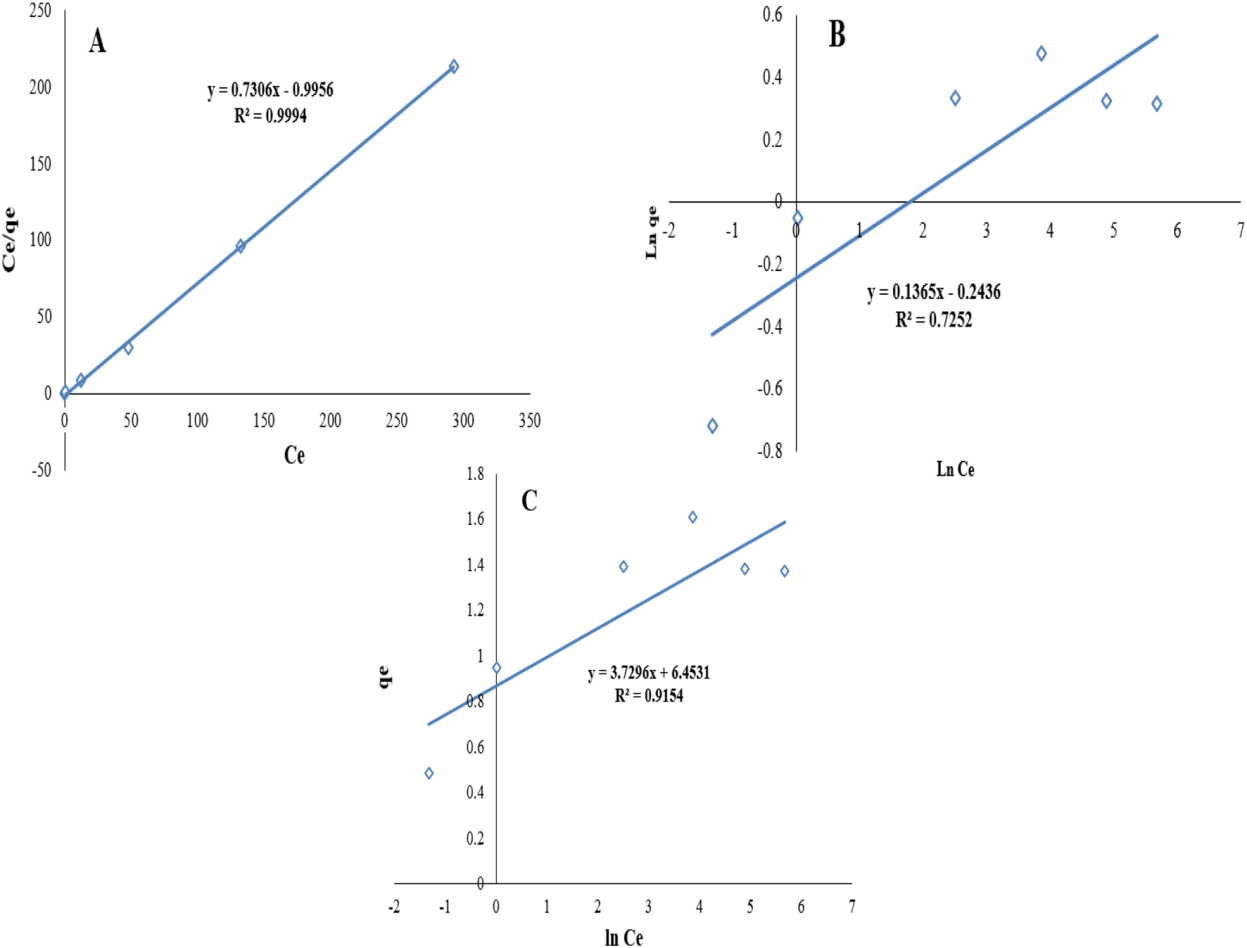
Adsorption isotherms for the removal of Cd by the MBA nano-hybrid: (**a**) Langmuir, (**b**) Freundlich and (**c**) Temkin (pH = 7.0, adsorbent = 1.0 g/L, temperature = 25 °C, optimum time = 60 min).

The isothermal curves constructed with the Langmuir, Freundlich, and Temkin correspond to a, b, and c in Fig. 10, respectively. Table 2 summarizes the calculated isothermal parameters. According to the results of Table 2, the maximum Cd(II) adsorption is approximately 35.36 mg/g at 25 °C. Compared with the Temkin and Freundlich models, the Langmuir isotherm model with R^2^ value of higher than 0.99 is more suitable for describing adsorption data of the Cd(II) adsorption on the MBA nano-hybrid.

**Table 2:** Isotherm modeling parameters for the removal of Cd by the MBA nano-hybrid: (a) Langmuir, (b) Freundlich (c) and Temkin. (pH = 7.0, adsorbent = 1.0 g/L, temperature = 25 °C, optimum time = 60 min).

Isotherm equation	Kinetic parameters	Values
Langmuir	q_m_(mg/g)	35.36
	b	0.73
	R_L_	0.026
	r^2^	0.99
Freundlich	*n*	7.32
	K_F_(mg/g(L/mg)^1/n^)	1.27
	r^2^	0.72
Temkin	A	3.72
	B	6.45
	r^2^	0.91

This result indicates uniform distribution of active sites on the MBA nano-hybrid surface, resulting in the single-layer adsorption of Cd(II) at homogeneous sites. Also, the adsorption of materials from the liquid phase onto the synthesized nano-hybrid is monolayer and the energy is the same during the adsorption process^42^. Also, in the Liu et al. study, which was performed to remove Cadmium, lead and copper ions from aqueous media, the results showed that the Langmuir model had a high regression coefficient compared with the Freundlich isotherm^46^. In the Badruddoza study, it was found that the adsorption data are more in line with the Langmuir isotherm, which is consistent with the results obtained from the present study^41^.

In addition, for further analysis of the Langmuir model, the dimensionless partition coefficient (R_L_) can be used (Eq. (10)), which is designed to evaluate the degree of adsorption desirability^7^. R_L_ > 1 indicates non-desirable type of adsorption, R_L_ = 1 shows linear adsorption, and 0 < RL < 1 demonstrates a desirable adsorption and an irreversible adsorption^47,48^.

10 $$R_{L} = \frac{1}{{1 + K_{L} c_{0} }}$$

The *R*_*L*_ value for the present study at 25 °C is 0.026, indicating an optimum adsorption of Cd(II) molecules on the MBA nano-hybrid. In the Freundlich model, on the other hand, the value of *n* is within the range of 1–10, indicating the desired adsorbent absorption on the adsorbent^7^.

Table 3 compares the maximum adsorption capacity (q_m_) of the MBA nano-hybrid for the adsorption of Cd(II) ions with other previously reported adsorbents. The data tabulated in Table 3 shows that the q_m_ of Cd(II) ions onto MBA nano-hybrid is higher than some of other adsorbents. The q_m_ value of MBA nano-hybrid adsorbing Cd(II) was higher than some of other adsorbents, and MBA could be recovered by a magnetic field, indicating that the adsorbent can be possibly applied for the removal of Cd(II) from aqueous solutions.

**Table 3:** Adsorption of Cd(II) ions from aqueous solutions by different adsorbents.

Adsorbent type	q_m_ (mg/g)	pH	Isotherm	Temperature (°K)	References
Attapulgite	10.38	5.45	L	298	^37^
Porous resin	3.51	7.0	L	298	^49^
Aluminum hydroxide modified steel-making slag	10.16	4.0	L	298	^50^
Montmorillonite	15.09	5.0	F	303	^51^
wheat straw	0.13	7.0	L	298	^52^
Bamboo charcoal	12.08	≥ 8.0	L	298	^53^
Modified sewage sludge	14.7	7.0	L	298	^54^
Bentonite	9.30	7.0	L	298	^55^
MBA nano-hybrid	35.36	7.0	L	298	This study

#### Effect Effect of coexisting cations

**Figure 11:**
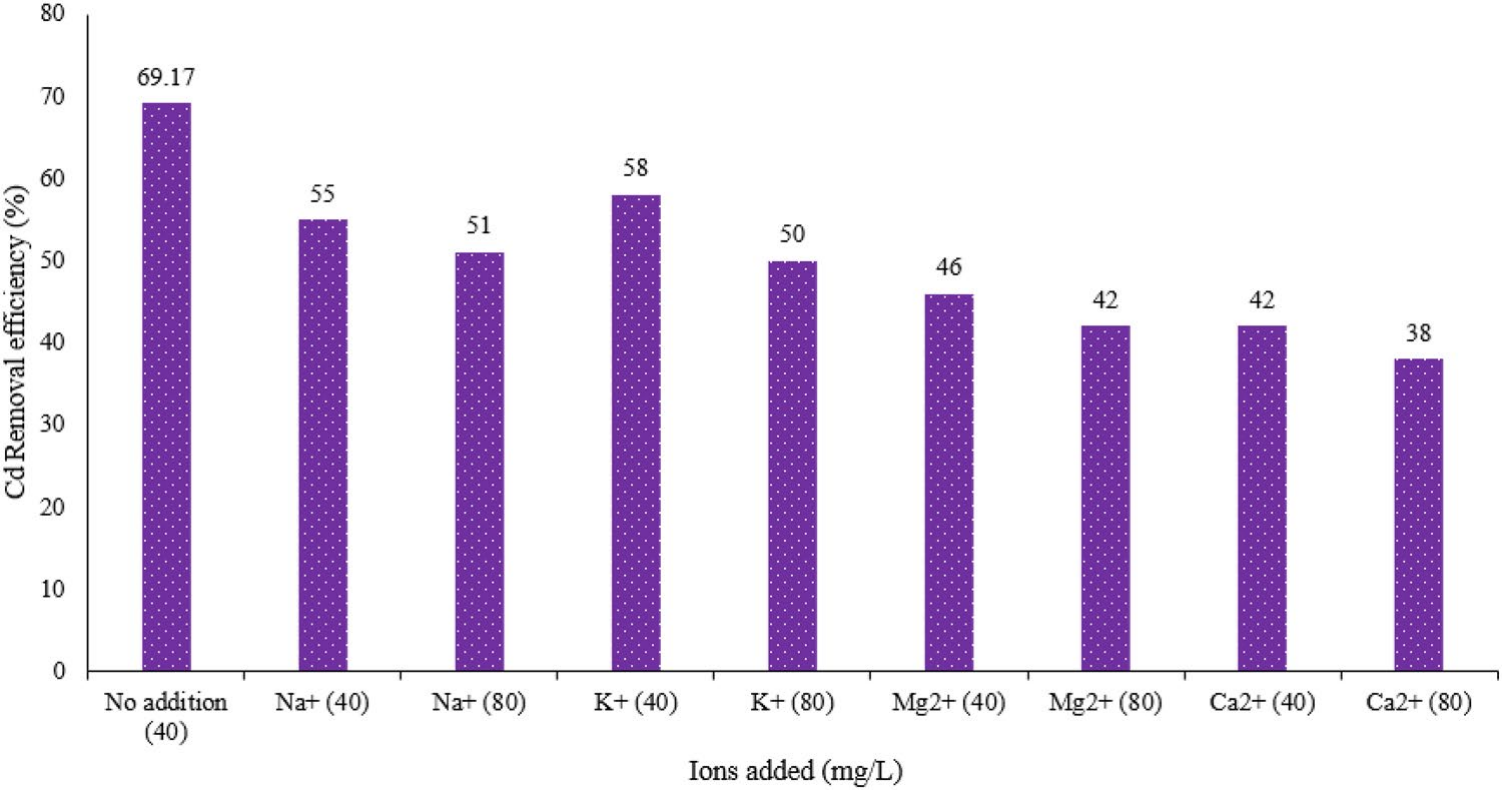
Effect of sodium, potassium, magnesium and calcium ions on the removal of Cd(II) by the MBA nano-hybrid (pH = 7.0, adsorbent = 1.0 g/L, temperature = 25 °C, time = 60 min).

The simulated test aqueous solution in this study does not contain other ions, while metal cations such as Ca^2+^, Mg^2+^, Na^+^ and K^+^ are very common in natural waters or actual wastewater and easily exhibit competitive adsorption with the target heavy metal ions^56^. In this study, the adsorption efficiency of Cd(II) ions on the MBA nano-hybrid was determined in the presence of four metal cations at two concentrations of 40 and 80 mg/L. As can be seen in Fig. 11, an increase in the concentration of metal cation ions from 40 to 80 mg/L results in a drop in Cd(II) removal efficiency.

The reduction in Cd(II) ions removal efficiency with the increasing the concentration of monovalent ions (Na^+^ and K^+^) had a slight inhibitory effect on the adsorption of Cd(II) ions, such that at a concentration of 80 mg/L in the presence of sodium and potassium ions, the removal efficiency dropped to 51 through 50% from 69.17% for the non-ionic increase. This is while the decrease in efficiency in increasing the concentration of divalent ions (Mg^2+^ and Ca^2+^) had a higher inhibitory effect on the adsorption of Cd(II) ions, such that at the 80 mg/L concentration in the presence of calcium and magnesium ions, the removal efficiency reduced to 42% through 38% from 69.17% for non-ionic enhancement. The greater effect of Ca^2+^ on Cd(II) adsorption is because of more similar ion radii of Ca^2+^ with Cd^2+^ than Mg^2+^ (ion radius are 0.99, 0.97 and 0.65 °A for Ca^2+^ with Cd(II) and Mg^2+^ respectively). On the other hand, Ca^2+^ can form complexes with water molecules, Ca^2+^-(H_2_O)_n_, that covering surface of adsorbent which resulting decreased availability of active sites on the adsorbent. It can be expected, Ca^2+^-(H_2_O)_n_ with larger radius than Mg^2+^-(H_2_O)_n_ has bigger steric hindrance and so its negative effect on adsorption is stronger^57,58^. Since concentration of Ca^2+^ and Mg^2+^ in fresh water is approximately between 10 and 80 mg/L, so the presence of these cations with these levels of concentration have not significant effect on Cd(II) adsorption.

#### Thermodynamic study

Solution temperature affects the rate of adsorption^59^. According to Arrhenius, absorption rate usually elevates with increasing temperature and reduces with decreasing temperature^33^. Such observation could be attributed to the increase in the size of the pores on the adsorbent surface or the increase in effective collisions between the adsorptive and the adsorbent^60^.

The adsorption mechanism for the Cd(II) adsorption on the MBA nano-hybrid is investigated by the thermodynamic parameters such as changes in enthalpy ΔH° (kJ/mol), entropy ΔS° (kJ/K.mol) and Gibbs free energy ΔG° (kJ/mol) based on the equations presented below [Eqs. (11)–(13)].

11 $$\Delta G^\circ = - RT (\ln Kc)$$

12 $$lnk_{c} = \Delta S^\circ /R - \Delta {\rm H}^\circ /RT$$

13 $$k_{c} = q_{e} /C_{e}$$

where *T* (°K) is the temperature, *R* (8.314 J/mol.K) is the global gas constant and *K*_*c*_ (L/g) is the ratio of the adsorbed material to the adsorbent remaining in the solution (mg/g).

The results of the thermodynamic study of the adsorption of Cd by the MBA nano-hybrid are presented in Table 4. As shown, the numerical value of ΔH° was obtained to be negative, indicating that the adsorption process is exothermic. In other words, the increase in temperature has a negative effect on the adsorption of Cd(II) and, the adsorption of this pollutant at lower temperatures is more favorable. The ΔS° parameter was positive in this process, showing an increase in the irregularity with increasing temperature in the solid–liquid phase during the adsorption process. A negative ΔG° also means that the adsorption process of Cd(II) on the adsorbent is feasible and thermodynamically spontaneous. These results are in agreement with the findings of Witek-Krowiak during the adsorption of heavy metals from aqueous solutions using beech wood sawdust^61^.

**Table 4:** Thermodynamic parameters for Cd(II) removal by the MBA nano-hybrid (pH = 7.0, adsorbent = 1.0 g/L, temperature = 25 °C, time = 60 min).

Concentration (mg/L)	ΔH°(kJ/mol)				ΔG° (kJ/mol)	ΔS° (kJ/K.mol)
	298 °k	308 °k	318 °k	318 °k		
40	− 8.13	− 7.16	− 6.06	− 5.54	88.21	− 20.88

#### Adsorbent reusability

**Figure 12:**
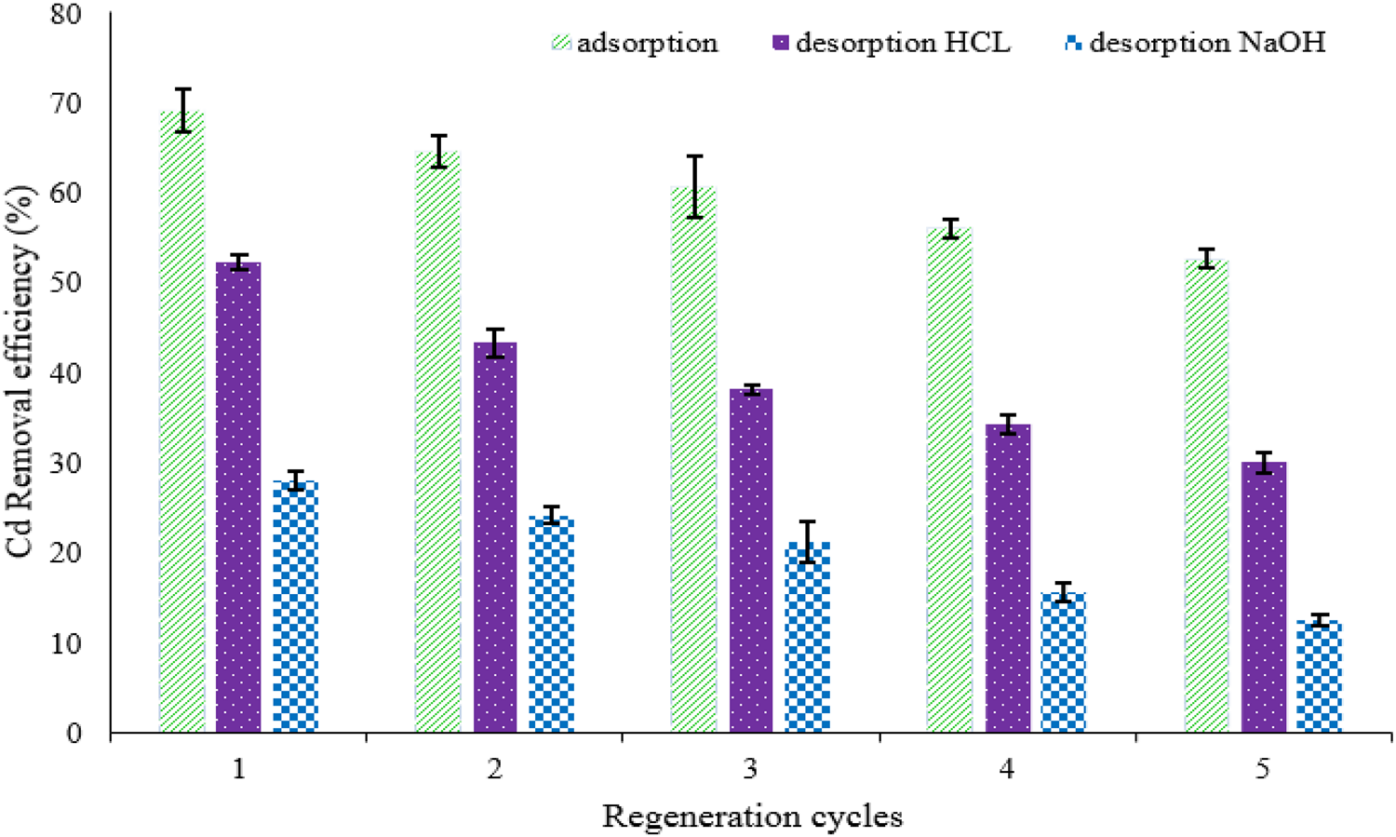
The removal efficiency of Cd(II) on fresh & regenerated MBA nano-hybrid with using HCL & NaOH. (pH = 7.0, adsorbent = 1.0 g/L, temperature = 25 °C, time = 60 min).

To investigate the reusability of adsorbent, MBA nano-hybrids were separated using an external magnetic field at the end of each adsorption run. Then, MBA nano-hybrids were placed into 20 mL of HCl (0.1 M) or NaOH (0.1 M) aqueous solutions as desorption solutions for 4.0 h, and washed three times with half-liter of D.I water to remove unadsorbed traces of Cd(II) ions. The washed and recovered adsorbent was dried at 60 °C for 24 h in an oven and reused in further adsorption cycles^62^. According to the results, the Cd(II) removal efficiency by the MBA nano-hybrid decreased from 69.17 to 52.6% within five adsorption cycles (Fig. 12). In other words, the MBA nano-hybrid can be recycled and reused for up to five consecutive times with the adsorption efficiency above 52.6%. According to the results, the desorption rate of HCl was higher than NaOH at all stages. When HCl was used as the desorption solution, the desorption percentage was greater than 52.1% for the Cd(II) adsorbed on the MBA nano-hybrid in the first stage and more than 30.1% at the end of fifth stage.

However, when NaOH was used as the desorption solution, the desorption rate was 28% for the adsorbed Cd(II) on the MBA nano-hybrid in the first stage and more than 12.5% in the fifth stage. The high amount of desorption using HCl can be attributed to the protonation of the adsorbent surface by the use of acidic agents. According to the Cechinel et al. study, HCl covers the surface of the adsorbent using H^+^ protons and completely protons the adsorbent for the next stage^63^. The results of the present study are also in agreement with those of Yi et al.^42,43^.

## Conclusion

In summary, the MBA nano-hybrid was synthesized and characterized for the adsorption of cadmium ion as target pollutant in the aquatic environment. According to the results, MBA nano-hybrid were successfully utilized for the adsorption of Cd(II) ions from aqueous solution. Moreover, the adsorption of Cd(II) ions onto the nano-hybrid was pH-dependent. The effect of contact time, initial Cd concentration, nano-hybrid dosage and temperature on the adsorption efficiency was fully evaluated. The adsorption data were well-fitted to the pseudo-second-order kinetic and Langmuir isotherm model with the maximum adsorption capacity of 35.36 mg/g. In the following, enthalpy, entropy and Gibbs free energy parameters of the Cd (II) ions adsorption were computed. Based on the activation parameters of the thermodynamic study, the adsorption of Cd(II) ions on the MBA nano-hybrid was spontaneous. The results of repeated adsorption–desorption cycles exhibited that MBA nano-hybrid can be effectively used as a recyclable adsorbent for the removal of Cd(II) from aqueous phase. Complementary experiments indicated that the as-prepared nano-hybrid can selectively adsorb Cd(II) ions in the presence of other competing cationic compounds. Conclusively, the MBA nano-hybrid can be proposed as an efficient adsorbent for treating Cd(II)-contained solutions. Actually, the results of the present study provide a promising treatment technique for the removal and also recovery of heavy metals from water and wastewater.

## Data Availability

The datasets used and/or analyzed in this study are available in the manuscript can be asked from the corresponding author upon request.
